# Social-media and newspaper reports reveal large-scale meteorological drivers of floods on Sumatra

**DOI:** 10.1038/s41467-020-16171-2

**Published:** 2020-05-19

**Authors:** Dariusz B. Baranowski, Maria K. Flatau, Piotr J. Flatau, Dwikorita Karnawati, Katarzyna Barabasz, Michal Labuz, Beata Latos, Jerome M. Schmidt, Jaka A. I. Paski, Marzuki

**Affiliations:** 1 Institute of Geophysics Polish Academy of Sciences, 64 Ksiecia Janusza, 01-452 Warsaw, Poland; 2 Naval Research Laboratory, 7 Grace Hopper Ave., Stop 2, Monterey, CA 93943-5502 USA; 3 Scripps Institution of Oceanography, University of California San Diego, 8622 Kennel Way, La Jolla, CA 92037 USA; 4 Agency for Meteorology, Climatology and Geophysics of the Republic of Indonesia (BMKG), Jl. Angkasa I No. 2, Kemayoran, Jakarta Pusat, 10720 Indonesia; 5 Collegium Civitas, Palace of Culture and Science, 12 floor, 1 Defilad Square, 00-901 Warsaw, Poland; 6 Michal Labuz, 30 Krakowska str., 34-730 Mszana Dolna, Poland; 7 Physics Department, Andalas University, Jl. Universitas Andalas, Limau Manis, Kec. Pauh, Kota Padang, Sumatera Barat 25163 Indonesia

**Keywords:** Atmospheric science, Hydrology, Environmental impact, Natural hazards, Developing world

## Abstract

Floods are a major contributor to natural disasters in Sumatra. However, atmospheric conditions leading to floods are not well understood due, among other factors, to the lack of a complete record of floods. Here, the 5 year flood record for Sumatra derived from governmental reports, as well as from crowd-sourcing data, based on Twitter messages and local newspapers’ reports, is created and used to analyze atmospheric phenomena responsible for floods. It is shown, that for the majority of analyzed floods, convectively coupled Kelvin waves, large scale precipitation systems propagating at ∼12 m/s along the equator, play the critical role. While seasonal and intraseasonal variability can also create conditions favorable for flooding, the enhanced precipitation related to Kelvin waves was found in over 90% of flood events. In 30% of these events precipitation anomalies were attributed to Kelvin waves only. These results indicate the potential for increased predictability of flood risk.

## Abstract (web summary)

Floods are an important natural disaster on the Indonesian island of Sumatra, but their driving mechanisms are not well understood. Here, the authors utilize data from twitter messages and local newspaper reports to show that convectively coupled Kelvin waves play a key role in promoting floods on Sumatra.

## Introduction

Flooding is one of the most frequent and damaging natural hazards globally, with numerous societal and economic effects. Between 2000 and 2018, meteorological and hydrological events accounted for 81.6% of all natural disasters, 78.0% of their losses and 27.9% of their fatalities^1^. The vulnerability of a population to adverse effects of a flood depends on natural conditions, as well as on the level of socioeconomic development—effects of natural hazards are more significant in relatively poor regions^2^. However, a recent report from the US National Academy of Sciences recognizes that urban flooding is a growing threat to even relatively wealthy municipalities in the United States, with higher risk to populations with less protection from insurance or the social safety net^3^. On the global scale, inhabitants of coastal cities in tropical regions are most vulnerable to flood damages, due to a combination of factors, both socioeconomic and natural^4^. Climate change is expected to make these conditions even worse^5–7^.

The Maritime Continent is an archipelago positioned within the Indo-Pacific warm pool and characterized by the largest average global precipitation, for which it has been referred to as the "boiler box” of the Earth^8,9^. The Indonesian (much of the Maritime Continent lies within the Republic of Indonesia) National Agency for Disaster Countermeasure (Badan Nasional Penanggulangan Bencana, BNPB) estimated that in 2017, floods accounted for more than 34% of all natural disasters nation-wide. While earthquakes and tsunamis are the most deadly Indonesian disasters, floods affected the lives of almost 14 million of Indonesian citizens between 1974 and 2013^10^, with a fatality rate from hydrological events exceeding 25 persons per 1M population^1^.

**Fig. 1:**
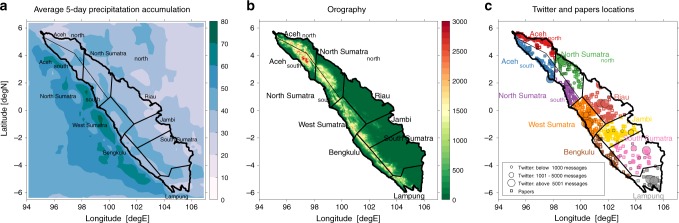
Environmental conditions and flood data availability in Sumatra. Map of Sumatra with division into provinces. The two most northern provinces (Aceh and North Sumatra) are additionally divided at the mountain range into a northern and southern part. **a** Average 5-day precipitation accumulation [mm] based on TRMM satellite-derived estimates. **b** Sumatra's topography [m]. **c** The spatial distribution of flood data sources from the Papers (squares) and Twitter (circles) in all of Sumatra's provinces between 2014 and 2018. Size of a circle represents the number of records at individual location. Each color shows one province.

The island of Sumatra is a northwest to southeast orientated land mass that straddles the equator on the western flank of the Maritime Continent adjacent to the Eastern Indian Ocean. Natural disasters in Sumatra threaten the livelihood of its over 50 million inhabitants. According to the BNPB, in one year alone (2017), 425,980 people were affected by floods. That year, flooding was responsible for about 50% of all natural disasters on the island, a percentage significantly higher than for all of Indonesia. It has been estimated that between 2010 and 2017, 87% of all natural disasters on Sumatra were weather-related^11^. While there are a number of other factors impacting the severity of a flood, including land use, human settlement patterns and catchments, precipitation intensity and accumulation are the most important and influence or interact with the other causes of flooding^12^. A typical 5-day precipitation accumulation over the island and its surroundings exceeds 30–50 mm (Fig. 1a). Therefore, understanding and predicting the variability of atmospheric circulation leading to convection and enhanced rainfall in such an extreme environment could help mitigate some of the adverse societal effects.

Precipitation within the Maritime Continent is driven by a very strong diurnal cycle of local convection^13–15^ and its variability in response to large scale modes such as the Madden Julian Oscillation (MJO) and equatorially-trapped waves such as convectively coupled Kelvin waves (CCKWs). The MJO is a repetitive large scale phenomenon extending over several thousand kilometers, which usually originates over the central Indian Ocean with intraseasonal (30–90 days) periodicity. While the MJO is a “convective envelope” that propagates eastward with a speed of about 5 m/s^16^, it can contain other convective disturbances, which can propagate either eastward (Kelvin waves) or westward (Rossby waves)^17,18^. CCKWs are also large scale phenomena but propagate much faster, with a speed of about 12 m/s, either within the MJO convective envelope or as separate modes^19–21^. CCKWs affect rainfall over the Maritime Continent through a coherent structure of anomalous precipitation with rates exceeding 10 mm/day^22,23^. Other significant modes responsible for enhancement of precipitation over the region include Boreal Summer Intraseasonal Oscillation during summer^24^ and the cold surges in the South China Sea in winter, especially when combined with the active phase of the MJO in the Indian Ocean^25^. The island of Sumatra, because of its orientation across the equator, is strongly impacted by tropical wave activity, especially eastward propagating modes (MJO and CCKW). These modes serve as a forcing mechanism for atmospheric convection and precipitation^15,22^, which can lead to floods^26^. Precipitation over Sumatra has a multi-scale character, with contributions from intraseasonal variability, convective super clusters, and diurnally forced orographic cloud systems^23,27^. It is worth noting, for example, that CCKWs have been recognized as one of the dominant factors influencing precipitation in other areas of the world, such as equatorial Africa^28,29^. We have also recently shown, through analysis of satellite precipitation data, that CCKWs have a direct impact on precipitation and diurnally forced convection over the Maritime Continent^22^. Therefore, CCKWs may be critical for flood prediction over Sumatra, and possibly for all of Indonesia as well.

The direct linkage between these large-scale equatorial disturbances and specific flooding events within the Maritime Continent or other regions has not been definitively established. This is an important linkage to demonstrate, as accurate forecasting of a flooding events can significantly decrease its societal impacts^30^. Currently, early warning systems in Indonesia rely on local real-time data sources: gauges and radar data. Such data combined with hydrologic models are valuable flood detection tools but have little potential for extended range forecasting: guidance which can provide critical lead-time to decision makers. Also, the data are sparse and often suffer from data gaps due to destroyed monitoring equipment.

In this study, we exploit the potential for predictability of floods in Sumatra based on their attribution to large-scale equatorially trapped meteorological phenomena. The study employs a synergistic methodology, utilizing flood data derived from governmental reports and crowd-sourcing (Twitter and local newspapers in Sumatra), as well as an analysis of meteorological data (local and large-scale precipitation and wind patterns). It is shown that a majority of floods in Sumatra are preceded by anomalous precipitation associated with CCKWs, which constitute the most important dynamical precursor for such events in the region. Therefore, weather services can leverage the predictability of floods by including tropical wave activity in their suite of operational weather forecast products.

## Results

### Flood data in Sumatra

Quantifying the relationship between eastward propagating organized convection and local hazards in Sumatra requires routinely available data about flooding events. Recently, the use of social media in detecting floods has shown promising results^31–33^ and could be used to fill these incomplete data gaps. This approach is resilient to extreme weather and, in fact, the quality of reports in both social and traditional media increases with the severity and adverse effects of a flood. Social-media based flood detection has been shown to be particularly useful in Indonesia^34^, where over 75% of active internet users are using Twitter, a social media platform. Nearly 12% of the total Indonesian population was active on Twitter in 2012 with the largest global tweet/user ratio at 1,813.53^35^. Information from local newspapers can also be mined and used to analyze the spatio-temporal distribution of floods. More importantly, the improved analysis of flooding acquired through social media provides a means to develop a direct correspondence between the flooding event itself and the larger-scale atmospheric phenomena from which it arise. It is this recognition which will lead to improved flood lead-time, both because these phenomena may be identified in advance of their passage and because of anticipated improvements in the numerically generated forecasted tracks and intensity. Therefore, in this study we establish the use of three independent datasets; two data bases that we developed for analyzing flood events based on Twitter messages and articles in local newspapers, and a third based on the Indonesian National Board for Disaster Management (BNPB) data. The data was grouped in space and time in order to allow a definite linkage between flood events and the large scale weather systems that set favorable flood conditions.

For the Twitter data base, all messages from the 2014–2018 period (hereafter tweets) referring to floods were processed with the TAGGS^34^ algorithm to geoparse the data and isolate those messages originating in Sumatra. Data from over 100 geotagged individual locations were binned to increase the number of available data around locations characterized by large Twitter activity (Fig. 1). Binning has been performed within Sumatra’s subregions, which match the borders of the island’s administrative provinces, except for two provinces in the northern part of the island: Aceh and North Sumatra. Those two provinces span the island and have significant topography which parallels the coast (mountain ranges with elevation exceeding 2500 m). Therefore, both Aceh and North Sumatra were further divided, along the mountain range, into two parts: north and south (Fig. 1b). As a result, 10 regions within Sumatra were identified and tweets were binned accordingly (Fig. 1). Aggregation of all available data from within a region potentially decreased the spatial resolution of the dataset to the size of a province. However, this was required to establish a reliable sample size for flood identification. This trade-off is justified, as the weather systems (MJO, CCKW) analyzed in this study have spatial scales much larger than a province.

**Fig. 2:**
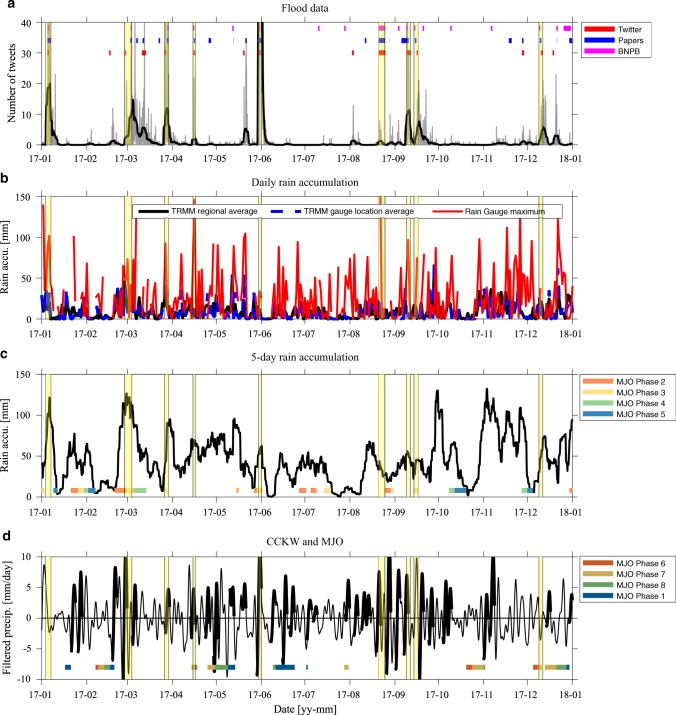
Floods and meteorological conditions in West Sumatra in 2017. Time series of flood and meteorological conditions during 2017 in West Sumatra. **a** 3-hourly Twitter data (gray line) and 3-day running average of Twitter data (black line); colored bars indicate floods identified in Twitter (red), paper (blue) and Indonesian National Board for Disaster Management (BNPB) (magenta) databases in 2017, (**b**) daily precipitation accumulation [mm] averaged over West Sumatra region from TRMM (black line), averaged over rain-gauge locations only from TRMM (blue dashed line) and maximum value of the three rain-gauges (red line), (**c**) 5-day precipitation accumulation [mm] averaged over the West Sumatra region in 2017 and Madden Julian Oscillation (MJO) phase Real-time Multivariate Madden Julian Oscilation index (RMM) phases 2–5 for RMM amplitude above 1 only, see legend), (**d**) convectively coupled Kelvin wave (CCKW) precipitation rate anomaly [mm/day] averaged over the the West Sumatra region and the MJO phase (RMM phases 6–1 for RMM amplitude above 1 only, see legend). Thick black lines in **d** indicate the robust CCKWs defined as the waves with a well defined trajectory^20^ at least between longitudes 80E and 110E. The yellow shading in all panels indicates major flooding periods for which identification from Twitter, Papers and BNPB agree.

Flood onset and termination times were identified automatically using a surge in tweets, that is, extremes in tendencies of tweet number time-series. Floods which occurred within 3 days of one another were considered part of a single event, a limit imposed by the minimal re-occurrence time of a CCKW^36^ and also close to the duration of the MJO precipitating phase affecting a single location^15^. Flood events identified using this technique match the rapid increase in tweets at the beginning of a flood (red lines in Fig. 2a, b for West Sumatra region).

Another source of crowd-sourced data—local papers—was analyzed to allow for cross validation with the Twitter database. These data should be considered more reliable than the Twitter data because newspaper reports are descriptive and include additional details and visual aids, such as photographs or links to videos showing conditions at a flood location. Therefore, they allow for the isolation of weather related floods. For consistency with the Twitter database, all individual papers’ reports were binned within the Sumatra regions shown in Fig. 1c. The third dataset utilized in this study is BNPB’s flood data base, which contains information on flooding events in Indonesia, including the Sumatra provinces. This dataset includes the start date of a flood alongside information about its adverse effects, such as number of people affected, damage to buildings and relief efforts. For consistency, both BNPB and the papers’ data were subject to the same 3-day re-occurrence threshold as the Twitter data. Thus, if 2 or more floods occurred within 3 days of one another, they were treated as a single event.

Identification of flood events based on the three independent databases agrees for most of Sumatra’s regions (Table 1). Exceptions are the Aceh and Bengkulu regions, where Twitter substantially underestimates (by a factor of 3) the number of floods in comparison with both the papers’ and BNPB databases, which agree with one another. On the other hand, in North Sumatra north and Lampung provinces, the number of floods identified in Twitter is larger (by a similar factor) than in other datasets. Interestingly, regardless of these differences, the seasonal cycle of floods (e.g., number of flood events during each season) is in agreement among all three databases. This indicates that biases in each dataset are random and independent of weather events or season. They likely result from factors such as regional variability of the population’s internet access, the increasing popularity of internet-based over traditional media (newspapers), and growing interest in floods and in improving the quality of their monitoring by governmental agencies. Synergistic analysis of all three databases mitigates some of these issues and allows independent quantification of large scale meteorological factors leading to floods.

**Table 1:** Number of floods in Sumatra according to the three database.

**Region**	**Twitter**		**Papers**		**BNPB**	
	**Tot**.	**DJF** **+** **MAM** **+** **JJA** **+** **SON**	**Tot**.	**DJF** **+** **MAM** **+** **JJA** **+** **SON**	**Tot**.	**DJF** **+** **MAM** **+** **JJA** **+** **SON**
Aceh north	14	7 + 0 + 0 + 7	43	16 + 8 + 6 + 13	42	16 + 4 + 4 + 18
Aceh south	38	11 + 5 + 5 + 17	59	15 + 13 + 9 + 22	69	15 + 17 + 12 + 25
North Sumatra north	71	19 + 13 + 12 + 27	49	13 + 12 + 4 + 20	52	15 + 10 + 4 + 23
North Sumatra south	23	8 + 3 + 1 + 11	31	8 + 9 + 1 + 13	25	6 + 6 + 2 + 11
Riau	92	28 + 19 + 12 + 33	59	18 + 15 + 6 + 20	37	13 + 8 + 2 + 14
West Sumatra	72	26 + 17 + 12 + 17	71	19 + 20 + 12 + 20	68	13 + 22 + 13 + 20
Jambi	30	17 + 6 + 1 + 6	38	21 + 10 + 0 + 7	35	18 + 9 + 3 + 5
Bengkulu	16	4 + 5 + 2 + 5	46	12 + 14 + 10 + 10	33	8 + 12 + 7 + 6
South Sumatra	49	18 + 13 + 4 + 14	38	14 + 13 + 3 + 8	53	22 + 19 + 3 + 9
Lampung	88	28 + 26 + 15 + 19	19	6 + 7 + 0 + 6	31	10 + 11 + 2 + 8
Entire Sumatra	493	166 + 106 + 65 + 156	453	142 + 121 + 51 + 139	445	136 + 118 + 52 + 139

The number of floods in 2014–2018 identified by tweets, the newspapers and Indonesian National Board for Disaster Management (BNPB) for ten Sumatra regions. Provided is total number (Tot.), as well as seasonal breakdown into boreal winter (DJF), spring (MAM), summer (JJA) and autumn (SON).

### Floods in the West Sumatra region

West Sumatra is positioned at the equator, right along the track of the eastward propagating large scale envelopes of organized convection, at the center of multi-scale interactions. In 2014–2018 it had more floods than any other region (Table 1). Fig. 2 shows results of flood identification and attribution to weather events for this region, based on three datasets. For clarity we show only one year, but it is representative of all years included in our analysis (2014–2018). In most cases, floods based on Twitter, papers and BNPB data match very well. Interestingly, some floods reported in the papers and Twitter are absent from BNPB’s dataset, whereas other events reported by BNPB are absent from the crowd sourcing data. Therefore, neither of those datasets can be considered a "gold standard” and using various independent sources of floods provides, in this case, more comprehensive information.

Nine major floods in West Sumatra, defined as events that appeared in all 3 databases, (yellow shading in Fig. 2) were identified for this period. Seven of these floods occurred during apparent spikes in precipitation measured by the three rain gauges located near Padang, the capital of West Sumatra. Rainfall accumulation, as expected, played a large role in the development of floods (Fig. 2c), confirming that these were in fact weather driven events. Eight of the major flooding events were associated with 5-day precipitation accumulation exceeding 50 mm. In seven of these events, the accumulation rapidly increased during or right before an event. During three of the major events, 5-day precipitation accumulation exceeded 90 mm. However, there were periods of such extreme precipitation accumulation associated with the reports in only one of the databases (e.g., newspaper database only) or no flooding reports at all. The maximum rainfall accumulation often occurred during MJO phases 2 and 3 (Figs. 2c and d), that is before the large scale MJO convective envelope reached Sumatra. That suggests a significant contribution to rainfall accumulation from local, diurnal convection which reaches a maximum when the MJO is located in the Indian Ocean and clear skies over the Maritime Continent allow large-scale heating of the land and development of intense afternoon precipitation^15^. Enhanced local precipitation can be also be related to higher frequency variability, such as CCKWs^22^ that can develop or propagate ahead of the MJO active phase.

Local in-situ and region-wide average precipitation in West Sumatra was associated with enhanced activity of CCKWs (Fig. 2b–d). Rain-gauge measured precipitation shows spikes (Fig. 2b) which correlate well with an increase both in precipitation accumulation (Fig. 2c) and in CCKW activity (Fig. 2d). Furthermore, the variability of satellite based precipitation estimates corresponds to in-situ data, but the values are substantially underestimated. This is a known caveat of satellite based estimates of surface precipitation, especially over complex topography^37^. On the other hand, frequent gaps in rain-gauge data (e.g., breaks in the red line in Fig. 2b) make them difficult to use for a long-term investigation of the variability of floods. Most of the 29 rain-gauge stations on Sumatra have 20–40% missing data during the 2014–2018 period of our analysis. Therefore, satellite based precipitation estimates are used in this study to evaluate regional precipitation, while keeping in mind that the local maxima may be underestimated.

CCKWs appear to have a strong influence on the timing of flooding events in West Sumatra. Six of the major events occurred during or immediately after a robust CCKW passage (Fig. 2d). We consider a CCKW robust if it has a precipitation anomaly of at least 2.5 mm/day and a well-defined equatorial trajectory^20^ between 80E and 110E (see Methods for a definition of CCKW trajectory). Since these disturbances propagate at a speed of about 10 degrees of longitude per day, well defined trajectories at 80E indicate that CCKWs related to floods were initiated at least two days before their arrival on Sumatra. Four of the major floods were associated with significant CCKW events, defined by a precipitation anomaly exceeding 5 mm/day. It is worth noting that while some of these waves existed within the propagating envelope of a MJO event, others were not associated with the active phase of intraseasonal variability. While the MJO has been recognized as a dynamical precursor for enhanced precipitation in Indonesia^15,27^, in West Sumatra, even during the MJO active phase (e.g., February–March 2017), a rapid increase in precipitation accumulation was associated with anomalous activity of CCKWs embedded in the MJO, as shown in both TRMM and rain-gauge data. On the other hand, a strong CCKW that developed outside of the MJO convective envelope (e.g., in September 2017) led to flooding, suggesting that CCKWs may play an important role in creating conditions favorable to enhanced precipitation and subsequent flooding, independently from other factors.

**Fig. 3:**
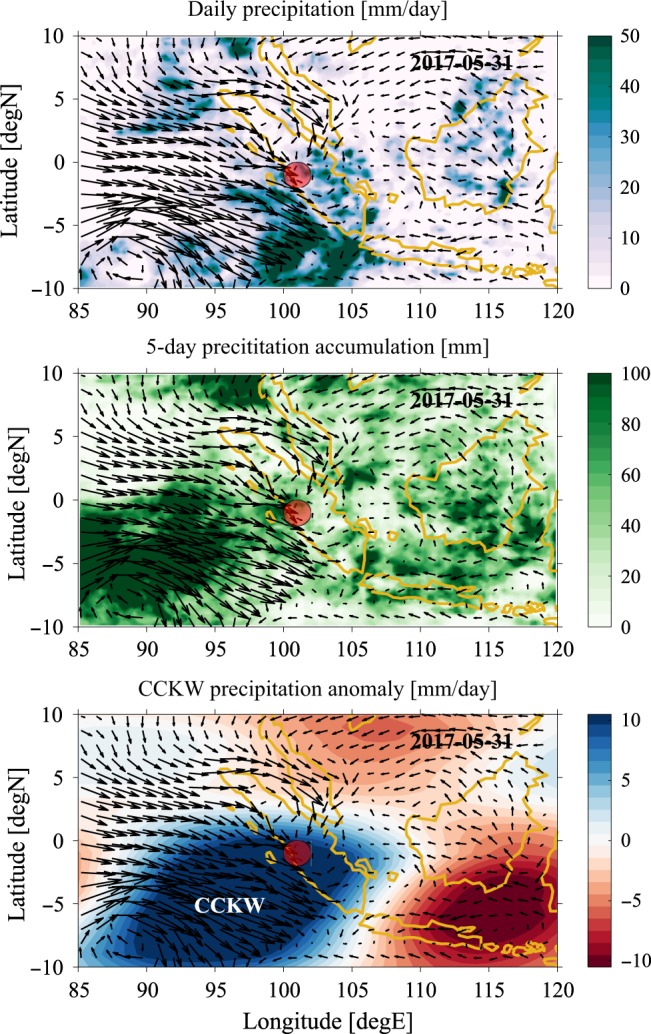
Meteorological conditions during Padang flood on 31 May 2017. Map of meteorological conditions on 31 May 2017. **a** the daily precipitation rate [mm/day], (**b**) 5-day precipitation accumulation [mm] and (**c**) convectively coupled Kelvin wave precipitation anomaly [mm/day]. Superimposed are 850 mb anomalous wind vectors. Marker indicates the Padang flood location.

**Fig. 4:**
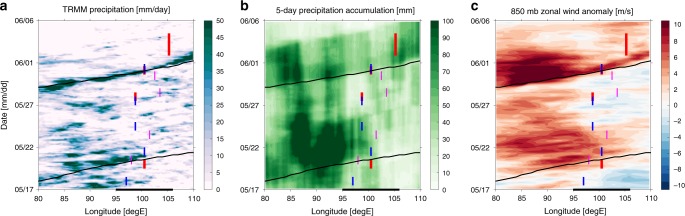
Meteorological conditions during Padang flood period. Hovmoller diagrams of meteorological conditions for periods 17 May–6 June 2017. **a** The 3-hourly precipitation rate [mm/day], (**b**) 5-day precipitation accumulation [mm] (**c**) 850 mb zonal wind anomaly [m/s]. All data are averaged in the 5S–5N meridional band. Superimposed are convectively coupled Kelvin wave trajectories (black lines). Colored lines indicate floods identified using Twitter (red), Papers (blue) and Indonesian National Board for Disaster Management (magenta) databases. The position of Sumatra is marked with a black bar on the bottom of the panel.

A case study of a major flood, which occurred near Padang (West Sumatra) on the 29 May–1 June 2017, is an example of an impact of eastward propagating convection on flooding conditions (Fig. 3 and Fig. 4). This flood was widely recognized in both social media (more than 1500 tweets throughout the event) and in local newspapers (reports in 5 papers), as well as in the BNPB database. Rain-gauge data from Padang and its surroundings (3 stations total) show maximum daily rainfall accumulation of 34 mm (Fig. 2b). Although this is substantial, it is far smaller than on many other days during that year, the vast majority of which did not exhibit any flooding (Fig. 2a). This illustrates the often non-local character of precipitation contributing to floods, especially in a complex topography, near valleys and rivers. Thus, in such cases precipitation averaged over a larger area is a better metric, and hence another advantage of satellite based estimates such as TRMM.

Preceding this flood, the MJO was active in the Indian Ocean (RMM phases 2 and 3). Although precipitation associated with this MJO envelope did not reach the MC, Sumatra was affected by a robust and strong CCKW (Fig. 4b–c) which had earlier passed through the MJO convective envelope. According to the CCKW trajectory data base, this wave originated over east Africa five days earlier and had a well-defined trajectory across the entire Indian Ocean.

This significant CCKW was associated with a precipitation anomaly exceeding 10 mm/day observed over Sumatra. Before May 31, enhanced precipitation was localized over the central and eastern Indian Ocean, resulting in a 5-day precipitation accumulation that exceeded 100 mm near Sumatra. (Fig. 3b and Fig. 4a). Unlike the MJO, in which the convectively active phase tends to damp local precipitation over land^38^, the convectively active phase of CCKW right over Sumatra is associated with the increase of precipitation and its diurnal maximum^22^. Therefore, anomalously heavy rain and 5-day accumulation of precipitation, which triggered flooding in Padang, was in this case primarily caused by the CCKW, likely enhanced by a favorable environment within the MJO envelope over the Indian Ocean. This event was also characterized by a strong low level westerly flow (Fig. 4c) that possibly enhanced local orographic precipitation. These anomalous westerlies were enhanced by Tropical Cyclone Mora, which formed on May 28th in the Bay of Bengal (88.5E, 14N) (http://www.rsmcnewdelhi.imd.gov.in/images/pdf/archive/bulletins/2017/nmora.pdf) and by cyclonic circulation that developed at 88E, 5S (Fig. 3) and was tracked by the Australian Bureau of Meteorology as low 31U. Such circulation is consistent with the structure of an equatorial Rossby wave^21^ or "cloud clusters”^17^, which can be triggered by an MJO itself^18,39^.

Overall characteristics of Sumatra floods

When considered as a whole, floods on Sumatra often occur simultaneously across multiple regions. Since our objective is to relate floods to large scale meteorological conditions, we focus on flooding periods defined here by a continuous sequence of floods occurring anywhere on Sumatra. During the 2014–2018 period, there were 493, 453, and 445 flooding events in individual regions in Sumatra, as identified in the Twitter, papers and BNPB databases, respectively (Table 1). Those events can be grouped into 254, 221, and 189 separate flooding periods affecting the entire island. The difference in the number of Sumatran floods reflects the random biases in the three datasets, as discussed before.

**Fig. 5:**
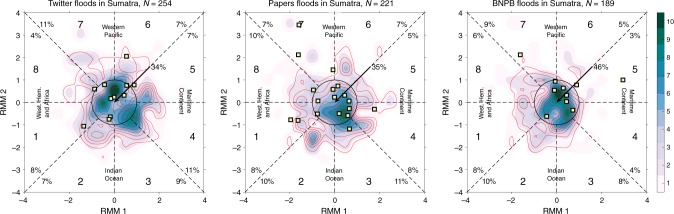
Aggregate floods on Sumatra in meteorological context. Flood periods in Sumatra between 2014 and 2018 from the Twitter (**a**), papers (**b**) and Indonesian National Board for Disaster Management (**c**) databases. Data presented on the RMM phase diagrams representing Madden Julian Oscillation (MJO) strength and location at the beginning of a flood. Shading indicates the number of floods associated with any convectively coupled Kelvin wave (CCKW) and contours indicate number of floods initiated during a strong CCKW event (contour interval is one). Rare floods occurring without any CCKW activity are marked with yellow squares. The MJO is considered active on days when its amplitude is above 1 (outside the unit circle) and location of active convection is represented in the phase. Phases 2 and 3 represent active MJO over Indian Ocean, phases 4 and 5 over the Maritime Continent, phases 6 and 7 over the Western Pacific and phases 8 and 1 over the Western Hemisphere and Africa. Percents indicates number of floods which occurred during each phase of the MJO and during no MJO activity. Total number of flood periods is indicated in the title of each panel.

About 34–46% of the floods (depending on the database) occurred during the time when the amplitude of the MJO index was less than 1, that is, when there was no MJO signal observed (Fig. 5). However, about 50% of the floods that developed during an active MJO (for RMM amplitude larger than 1) occurred when the intraseasonal convection was away from the Maritime Continent (phases 6–1), which means that locally over Sumatra, the MJO related precipitation was suppressed. Only 28–34% of all flooding periods on Sumatra occurred during MJO conditions potentially favorable for enhanced precipitation (phases 2–5) over Indonesia. Since Sumatra is on the western edge of the Maritime Continent, the MJO location over the Indian Ocean (phase 2–3) is considered to be a better predictor of intense precipitation over the island than MJO phases 4–5. However, less than 23% of all flooding periods began when the MJO was active over the Indian Ocean (phases 2–3).

On the other hand, over 91% of flooding period onsets were associated with some CCKW activity at 90E with a precipitation anomaly exceeding 2.5 mm/day) and over 63% began during strong CCKWs (Fig. 5). The CCKW trajectory analysis, which is constrained to the equator, shows that 34% or more of flooding period onsets were associated with a robust trajectory. Therefore, it appears that CCKWs provide favorable conditions for flood development for the entire island. In fact, about 45% (66–79 out of 160 events) of robust CCKW trajectories were associated with flooding periods on Sumatra. This analysis also shows relative differences between databases. For example, unlike the BNPB database, Twitter and papers show a significant number of floods initiated during MJO active phases 3–5. On the other hand, for MJO amplitude of less than one (within the circle in Fig. 5), the distribution of flooding periods and their relation to CCKW activity is more consistent between BNPB and Twitter databases, while papers paint a slightly different picture. Therefore, comprehensive analysis of the three data sources provides more reliable information about floods and their relationship with large scale weather.

## Discussion

Extreme tropical precipitation and the flooding that results from it are associated with various modes of atmospheric variability. The island of Sumatra, where in 2017 rainfall related events accounted for more than 50% of natural disasters, is characterized by extremely high average precipitation accumulation (Fig. 1a). Rainfall on Sumatra is dominated by a strong diurnal cycle of convection^13^, which develops over steep topography^27,40^ and its interaction with large scale equatorial disturbances. In this study we have shown that one class of these modes—convectively coupled Kelvin waves—constitutes a critical dynamical predictor for Sumatran flood onsets. We also demonstrated the usefulness of social media-based methods of flood detection in supplementing other information about flood occurrences and in increasing the reliability of the attribution of floods to meteorological phenomena.

**Fig. 6:**
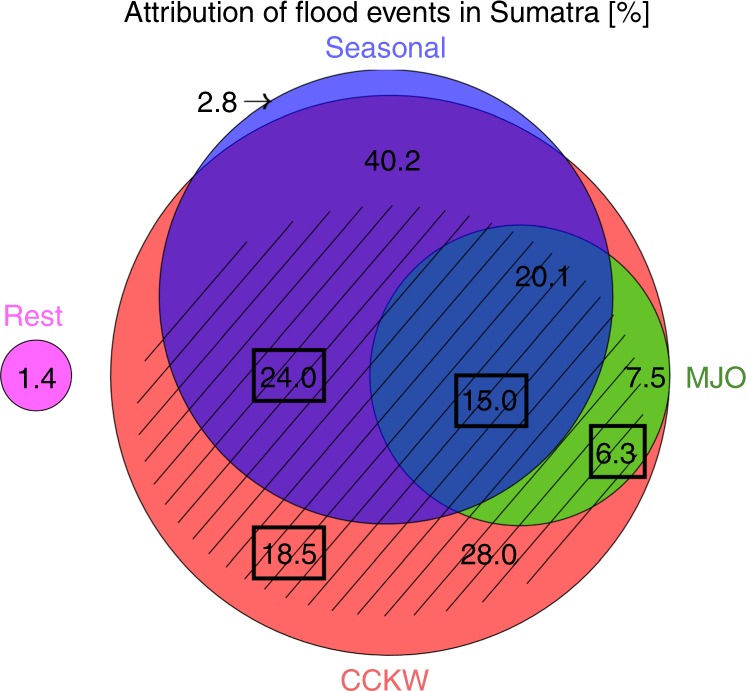
Attribution of flood events in Sumatra. Data presented as scaled Venn diagram. Total area of the blue circle represents 63.1% of floods associated with above-average seasonal precipitation. Total area of the green circle represents 27.6% floods associated with favorable Madden Julian Oscillation (MJO) conditions MJO in phases 2–5). Total area of the red circle represents 95.8% floods associated with convectively coupled Kelvin wave (CCKW) activity (precipitation anomaly above 2.5 mm/day), while hatching indicates strong CCKWs (precipitation anomaly above 5 mm/day). Numbers indicate percentage of floods in respective categories. Numbers in boxes represent strong CCKWs. The magenta circle represents 1.4% of all floods, which were not attributed to either category considered. The diagram is based on Twitter database.

The study is based on a synergistic methodology employing multi-year analysis of meteorological data (precipitation, surface winds) and three independent datasets of floods, in order to consistently analyze spatio-temporal variability in flooding events and the environmental conditions leading to them. Two flood databases were derived from crowd-sourcing: Twitter and local newspapers. Tweets related to floods were identified using keywords, geoparsed to select messages, which originated in Sumatra and binned within Sumatra’s sub-regions. Similar binning was performed on the data from local papers. Additionally, the database from the Indonesian agency BNPB was used. All three datasets were independently analyzed. While not all floods are identified in every database, the results obtained from our analyses agree for all key elements of this study, providing confidence in our findings. Indonesia, due to the large number and strong activity of Twitter users, is perfect for such a study, but this approach can be utilized in other regions having sufficiently high activity of social media users. The increasing popularity of various social media platforms should allow future studies which focus on longer periods of time, as well as on other meteorological phenomena, worldwide (Fig. 6).

shows the attribution of flooding to various modes of variability on Sumatra. Seasonal variability in precipitation and flood occurrence (Table 1) is associated with monsoonal flow. Namely, regions in the northern part of the island most frequently suffer from floods during boreal fall and winter, whereas regions in the southern part are mostly affected during boreal winter and spring. Flooding in the central part of Sumatra occurs most frequently during fall and spring. No region in Sumatra exhibits maximum flood intensity during boreal summer. This seasonal variability in flood frequency is well explained by the annual progression of the Inter-Tropical Convergence Zone. Such climatological analysis provides some information about the probability of flood occurrence, but does not prove useful in short to mid-range flood forecasting.

On a synoptic time scale, the amount of rainfall over the island is variable as well and strongly modulated by eastward propagating modes of organized convection: the MJO^15^ and CCKWs^22^, both of which affect the local diurnal cycle through multi-scale processes. Although our results recognize some importance of the MJO as indicated by previous studies^23,25^, we find that only about 28% of floods in Sumatra were immediately preceded by favorable MJO conditions and all of those events, similarly to the Padang flood discussed in this paper, were in fact triggered by CCKWs embedded within the envelope of enhanced MJO convection (Fig. 6).

Comprehensive analysis shows that about 45% of robust CCKW events, which propagated between 80E and 110E, were associated with floods in Sumatra. From a different perspective, about 60% of floods in Sumatra were immediately preceded by a robust CCKW event. This percentage is substantially higher than flooding events associated with favorable MJO conditions, indicating stronger interaction of local convection with CCKWs. Based on that analysis, nearly half of Sumatran flooding periods had a well-defined dynamical precursor at least two days before flood onset, a fact that can be utilized to improve warning lead times.

Our estimates of the role of CCKWs in triggering floods are conservative. We believe that the impact of CCKWs may be even larger than what is shown in this paper. One reason is that the CCKW trajectories used in this investigation are confined to the equator, while in reality Kelvin waves propagate along the "dynamical equator", defined by zero ambient absolute vorticity which may be displaced from the geographic equator. Therefore our scheme may be missing some of the off-equatorial Kelvin waves and their impact on floods. Thus, the obtained results constitute a lower boundary estimate of the influence of CCKWs on floods on Sumatra. In addition, we considered the influence of CCKWs on flood initiation only, and not on the enhancement of precipitation once the flood is already detected. That could also contribute to an under-estimation of CCKW impact.

Although the period investigated in this study (2014–2018) is relatively short, the remarkable agreement between the three independent databases demonstrates the robustness of its key findings. In the future, the volume of crowd-sourced data, efficient data mining and processing methods should allow for better parameters for flood detection, taking into account regionally specific characteristics of social media users.

While the focus of this paper is on the role of Kelvin waves, in future work the interaction between various equatorial modes should also be considered. For example, the interaction of Kelvin waves with off-equatorial vortices (shown in the case study for 31 May 2017 discussed in this paper) could be an important factor in creating strong cross-barrier westerlies which interact with the island orography. In fact in some flow regimes, vortices could be triggered by the Sumatran topography^41^. Equatorial Rossby waves can also originate within region of active intraseasonal convection^18,39^, and intense vortices can be generated by the Kelvin wave itself^42^. In either case, equatorial westerlies are enhanced.

A thorough understanding of the role of the MJO and other equatorial modes in Sumatran floods creates the opportunity for better prediction. Recent advancements in modeling^43,44^ have contributed to increased forecast skill on subseasonal time scales. The prediction of CCKWs is still challenging, but improvements in convective parameterizations have led to more skillful forecasts^45^.

Since CCKWs provide additional predictability in the short term (currently 3–5 days), both the MJO and CCKWs can be leveraged to improve forecasting of flooding events in Sumatra and potentially in other parts of the Maritime Continent. Given Sumatra’s extreme conditions which impacts the vulnerability of its inhabitants, such predictability should be exploited in order to mitigate the socioeconomic costs of floods.

## Methods

### Twitter processing

All tweets for the 2014–2018 period referring to floods (Indonesian: "banjir") were identified. These messages were geoparsed using the TAGGS algorithm^34^ to allow for identification of data originating in Sumatra. Although tweet metadata can explicitly include geographical location, most users choose not to share these data. The TAGGS algorithm allows geoparsing based on other metadata, such as the user’s nationality and home town, as well as direct referrals to specific locations (e.g., name of the province or city) in the message itself. Although this method is an estimation and may result in parsing a message to an inaccurate location, this study relies on a large number of tweets for identification of each flood event. Using a large quantity of data, rather than single tweets, minimizes random noise due to the potentially inaccurate geoparsing of some messages. All Twitter data for Sumatra were then binned and processed. Regional data were binned to a 3-hourly temporal grid (e.g., gray line in Fig. 2a) and flooding events were identified based on a surge in tweet numbers. To exclude high frequency noise, the time series were low-pass filtered with a 3-day running average (black line in Fig. 2a) and local extrema were identified. A local maximum associated with a number of tweets above a certain, region-specific threshold was considered the start of a flood. If such maximums occurred within three days of one another, they were considered part of a single event. The first local minimum following the last maximum in a sequence was considered the end of an event. The threshold (noise) level was calculated for each region independently, based on overall Twitter activity within that region. This was determined by the 85th percentile of 30-day low-pass filtered Twitter activity distribution. Such a definition allows for region-specific determination of adequate noise level based on overall Twitter activity and satisfies criteria that floods are rather rare, rapid and extreme events.

### Meteorological data

The meteorological data used in this study include precipitation estimates from Tropical Rainfall Measuring Mission (TRMM) and Global Precipitation Measurement (GPM) satellite missions and their derivatives, rain gauge data from Sumatra, 850mb zonal winds from ECMWF’s ERA5 reanalysis and state of intraseasonal variability as assessed by Realtime Multivariate MJO index (RMM)^46^. This index provides information about the strength and location of intraseasonal convection associated with the MJO, referred to as amplitude and phase, respectively. If the amplitude exceeds 1, the MJO is considered active in a given phase. Phases 2 and 3 indicate intraseasonal convection located over the Indian Ocean and phases 4 and 5 indicate its position over the Maritime Continent.

The precipitation estimates used in this study come from a gridded 3B42v7 product (TRMM (TMPA) Rainfall Estimate L3 3-hour 0.25 Degree × 0.25 Degree V7, DOI:http://dx.doi.org/10.5067/TRMM/TMPA/3H/7)^47^, which utilizes both TRMM and GPM measurements. It provides 3-hourly maps on a 0.25^∘ ^× 0.25^∘^ grid. The 5-day precipitation accumulation was calculated on the same grid. In addition, in-situ rain gauge data from three available stations near Padang, West Sumatra, were used (one meteorological station, one climatological station and one geophysical station). The data was obtained from an online database developed by the Agency for Meteorology, Climatology and Geophysics of the Republic of Indonesia (BMKG) http://dataonline.bmkg.go.id/(accessed: 09/23/2019). Apart from the operational quality control of the rain gauge data applied by BMKG, no additional quality control was applied.

Precipitation anomalies associated with CCKWs were calculated using Fourier space wavenumber-frequency filtering^48^. In addition, CCKW trajectories were calculated based on filtered precipitation anomalies within the equatorial band (2.5S–2.5N)^20^.The method tracks anomalies that are continuous in space and time locally exceeding a threshold value of 2.5 mm/day.

Seasonal precipitation over Sumatra has been calculated based on an area-averaged 5-day precipitation accumulation estimate from TRMM3B42v7. A 90-day running average low pass filter was applied to the time series to extract the seasonal cycle of precipitation. On days when the index exceeded its 5-year-mean value, seasonal conditions were considered favorable for flooding.

It should be noted that over steep orography, the TRMM3B42v7 product tends to underestimate the precipitation rate^37^. Therefore, the precipitation accumulation and CCKW anomaly over the islands, especially over the mountains near the west coast of Sumatra, should be considered a lower boundary estimate.

The ERA5 hourly zonal winds at 850 mb^49^ were averaged to 3-hourly to match the resolution of the TRMM data. They are presented as anomalies with the seasonal cycle removed. The seasonal cycle was derived by fitting the first three annual harmonics to a multiyear (2000-2018) average annual cycle.

Consistency between flood databases and meteorological data in regional analysis was achieved by averaging meteorological data (precipitation accumulation and CCKW precipitation anomaly) over respective regions. Additionally, for CCKW trajectory analysis, the base point was chosen at 90 East over the eastern Indian Ocean. Over the Indian Ocean, the CCKWs tend to move eastward along the equator. They arrive on Sumatra about one day later. CCKW trajectories provide constraints on the onset and decay of the location of a particular event. In this study, robust CCKW trajectories are identified if they are continuous between 80E and 110E, which means that they were initiated at least 2 days before approaching Sumatra and being propagated across the island.

## Data Availability

All relevant data are available from the authors through the corresponding author. Precipitation data from rain gauges were obtained from an online database (http://dataonline.bmkg.go.id/) supported and maintained by the Indonesian Meteorological, Climatological, and Geophysical Agency. Satellite based precipitation estimates were obtained from the Precipitation Processing System’s FTP server (http://ftp://trmmopen.gsfc.nasa.gov/pub/merged/3B42/). Wind data were downloaded from the Copernicus Climate Change Service Climate Data Store (https://cds.climate.copernicus.eu/cdsapp#!/home).

## Code availability

The MATLAB code used for processing the Twitter and papers’ data is available upon request.

## References

[1] MunichRe. NatCatSERVICE Database (Munich Reinsurance Company, Geo Risks Research, Munich). https://natcatservice.munichre.com/. Accessed 8 May 2019 (2019).

[2] Jongman B (2015). Declining vulnerability to river floods and the global benefits of adaptation. Proc. Natl Acad. Sci. USA.

[3] National Academies of Sciences & Medicine. *Framing the Challenge of Urban Flooding in the United States.* https://www.nap.edu/catalog/25381/framing-the-challenge-of-urban-flooding-in-the-united-states. (The National Academies Press, Washington, DC, 2019). 31091058

[4] Hallegatte S, Green C, Nicholls RJ, Corfee-Morlot J (2013). Future flood losses in major coastal cities. Nat. Clim. Change.

[5] Kundzewicz ZW (2014). lood risk and climate change: global and regional perspectives. Hydrological Sci. J..

[6] Feng X, Porporato A, Rodriguez-Iturbe I (2013). Changes in rainfall seasonality in the tropics. Nat. Clim. Change.

[7] Hirabayashi Y (2013). Global flood risk under climate change. Nat. Clim. Change.

[8] Ramage CS (1968). Role of a tropical "Maritime Continent” in the atmospheric circulation. Monthly Weather Rev..

[9] Simpson J, Keenan TD, Ferrier B, Simpson RH, Holland GJ (1993). Cumulus mergers in the Maritime Continent region. Meteorol. Atmos. Phys..

[10] Hapsari RI, Zenurianto M (2016). View of flood disaster management in Indonesia and the key solutions. Am. J. Eng. Res..

[11] Indonesian National Board for Disaster Management (Badan Nasional Penanggulangan Bencana, BNPB). http:/bnpb.cloud/dibi/tabel1b. Accessed10 May 2019 (2019).

[12] Pramono, I. & Savitri, E. Flash flood in Arau watershed, West Sumatera: a mitigation study. In *MATEC Web of Conferences*, Vol. 229, 03002 (EDP Sciences, 2018).

[13] Qian J-H (2008). Why precipitation is mostly concentrated over islands in the Maritime Continent. J. Atmos. Sci..

[14] Yamanaka, M.D. et al. Maritime Continent coastlines controlling Earth’s climate. *Progress Earth Planetary Sci.* **5**, 21 (2018).

[15] Peatman SC, Matthews AJ, Stevens DP (2014). Propagation of the Madden–Julian oscillation through the Maritime continent and scale interaction with the diurnal cycle of precipitation. Q. J. Roy. Met. Soc..

[16] Zhang, C. Madden-Julian oscillation. *Rev. Geophys.* **43**, RG2003 (2005). 10.1029/2019RG000685 PMC7375192 32879923

[17] Nakazawa T (1988). Tropical super clusters within intraseasonal variations over the Western Pacific. J. Meteorological Soc. Jpn. Ser. II.

[18] Ferreira RN, Schubert WH, Hack J (1996). Dynamical aspects of twin tropical cyclones associated with the Madden-Julian Oscillation. J. Atmos. Sci..

[19] Roundy, P. E. Analysis of Convectively Coupled Kelvin waves in the Indian Ocean MJO. *J. Atmospheric Sci.* **65**, 1342–1359 (2008).

[20] Baranowski DB, Flatau MK, Flatau PJ, Matthews AJ (2016). Impact of atmospheric convectively coupled equatorial Kelvin waves on upper ocean variability. J. Geophys. Res..

[21] Kiladis, G. N., Wheeler, M. C., Haertel, P. T., Straub, K. H. & Roundy, P. E. Convectively coupled equatorial waves. *Rev. Geophys.* **47**, RG2003 (2009).

[22] Baranowski DB, Flatau MK, Flatau PJ, Matthews A (2016). Phase locking between atmospheric convectively coupled equatorial Kelvin waves and the diurnal cycle of precipitation over the Maritime Continent. Geophys. Res. Lett..

[23] Shibagaki Y (2006). Multiscale aspects of convective systems associated with an intraseasonal oscillation over the Indonesian Maritime Continent. Monthly Weather Rev..

[24] Faqih, A. & Nurussyifa, D. Intraseasonal rainfall variability in North Sumatra and its relationship with Boreal Summer Intraseasonal Oscillation (BSISO). In *IOP Conference Series: Earth and Environmental Science*, Vol. 54, 012033 (IOP Publishing, 2017).

[25] Fauzi, R. & Hidayat, R. Role of cold surge and MJO on rainfall enhancement over Indonesia during East Asian winter monsoon. In *IOP Conference Series: Earth and Environmental Science*, Vol. 149, 012045 (IOP Publishing, 2018).

[26] Wahyuni S, Marzuki, Pujiastuti D, Sani LF, Rahayu A (2015). Review of meteorological flood conditions for the Padang on 24 July 2012 (in Indonesian). J. Fis. Unand.

[27] Marzuki (2013). Cloud episode propagation over the Indonesian Maritime Continent from 10 years of infrared brightness temperature observations. Atmos. Res..

[28] Schlueter A, Fink AH, Knippertz P, Vogel PA (2019). Systematic comparison of tropical waves over Northern Africa. Part I: Influence on Rainfall. J. Clim..

[29] Nguyen, H. & Duvel, J.-P. Synoptic wave perturbations and convective systems over equatorial Africa. *J. Clim.* **21**, 63726388 (2008).

[30] Karnawati D (2018). Foreword by Dwikorita Karnawati for the Journal of the International Consortium on Landslides. Landslides.

[31] Wang R-Q, Mao H, Wang Y, Rae C, Shaw W (2018). Hyper-resolution monitoring of urban flooding with social media and crowdsourcing data. Computers Geosci..

[32] Jongman, B. Effective adaptation to rising flood risk. *Nat. Commun*. **9**, 1–3 (2018). 10.1038/s41467-018-04396-1 PMC5974412 29844334

[33] Holderness, T. & Turpin, E. From social media to geosocial intelligence: crowdsourcing civic co-management for flood response in Jakarta, Indonesia. In *Social Media for Government Services*, 115–133 (Springer, 2015) .

[34] de Bruijn, J. A., de Moel, H., Jongman, B., Wagemaker, J. & Aerts, J. C. TAGGS: grouping tweets to improve global geoparsing for disaster response. *Geovisualizat. Spatial Anal.* **2**, 2 (2018).

[35] Carley, K.M., Malik, M., Kowalchuck, M., Pfeffer, J. & Landwehr, P. Twitter usage in Indonesia. Available at SSRN: https://ssrn.com/abstract=2720332 (2015).

[36] Baranowski, D.B., Flatau, M.K., Flatau, P.J. & Schmidt, J.M. Multiple and spin off initiation of atmospheric convectively coupled Kelvin waves. *Clim. Dynamics* **49**, 29913009 (2017).

[37] Matthews AJ, Pickup G, Peatman SC, Clews P, Martin J (2013). The effect of the Madden-Julian oscillation on station rainfall and river level in the Fly River system, Papua New Guinea. J. Geophys. Res.

[38] Birch CE (2016). Scale Interactions between the MJO and the Western Maritime Continent. J. Clim..

[39] Liebmann B, Hendon HH, Glick JD (1994). The relationship between tropical cyclones of the western Pacific and Indian oceans and the Madden-Julian Oscillation. J. Meteorological Soc. Jpn. Ser. II.

[40] Nesbitt SW, Zipser E (2003). The diurnal cycle of rainfall and convective intensity according to three years of TRMM measurements. J. Clim..

[41] Fine CM, Johnson RH, Ciesielski PE, Taft RK (2016). The role of topographically induced vortices in tropical cyclone formation over the Indian Ocean. Monthly Weather Rev..

[42] Schreck III CJ (2015). Kelvin waves and tropical cyclogenesis: a global survey. Monthly Weather Rev..

[43] Kim H, Vitart F, Waliser DE (2018). Prediction of the Madden-Julian oscillation: a review. J. Clim..

[44] Janiga MA (2018). Subseasonal forecasts of convectively coupled equatorial waves and the MJO: activity and predictive skill. Monthly Weather Rev..

[45] Dias J (2018). Equatorial waves and the skill of NCEP and ECMWF numerical weather prediction systems. Monthly Weather Rev..

[46] Wheeler MC, Hendon HH (2004). An all-season real-time multivariate MJO index: development of an index for monitoring and prediction. Monthly Weather Rev..

[47] Huffman GJ (2007). The TRMM Multisatellite Precipitation Analysis (TMPA): Quasi-Global, multiyear, combined-sensor precipitation estimates at fine scales. J. Hydrometeorol..

[48] Wheeler M, Kiladis GN (1999). Convectively coupled equatorial waves: analysis of clouds and temperature in the wavenumber-frequency domain. J. Atmos. Sci..

[49] Copernicus Climate Change Service (C3S). ERA5: Fifth generation of ECMWF atmospheric reanalyses of the global climate. https://cds.climate.copernicus.eu. Accessed 8 May 2019 (2017).

